# Optimizing Nutrition in Renal Patients: Effects of a Low-Protein Diet Supplemented With Ketoacids

**DOI:** 10.7759/cureus.38205

**Published:** 2023-04-27

**Authors:** Roy Aghwana, Henry O Aiwuyo, Henry Ovwasa, Ogochukwu Okoye, Anthony G Kweki, Evelyn Unuigbe

**Affiliations:** 1 Internal Medicine, Colchester General Hospital, Colchester, GBR; 2 Internal Medicine, Brookdale University Hospital Medical Center, Brooklyn, USA; 3 Family Medicine, Milk River Health Center, Alberta, CAN; 4 Internal Medicine, Delta State University Teaching Hospital (DELSUTH), Oghara, NGA; 5 Internal Medicine/Cardiology, Colchester General Hospital, Colchester, GBR; 6 Internal Medicine, University of Benin Teaching Hospital, Benin City, NGA

**Keywords:** nutritional indices, ketoacids, low protein diet, renal patient, nutrition

## Abstract

Background

Chronic kidney disease (CKD) is a non-communicable disease; it is a major cause of morbidity and mortality in Nigeria as the incidence has been increasing in Nigeria over the last few years. A low-protein diet supplemented with ketoacids has been duly documented to reduce the malnutrition associated with CKD as well as improve estimated glomeruli filtration rate while delaying the onset of dialysis in predialysis CKD patients.

Objective

The aim of this study was to determine the effects of a low-protein diet supplemented with ketoacids compared to a conventional low protein on nutritional indices in predialysis CKD patients.

Methods and materials

A randomized controlled trial with a total of 60 participants was conducted at Delta State University Teaching Hospital (DELSUTH), Oghara, Nigeria. Participants were patients older than 18 years with CKD stage 3-5 who were not on dialysis. They were recruited and randomized into the intervention group (low-protein diet supplemented with ketoacids) with 30 participants and the non-intervention group (low protein with placebo) with 30 participants. The mean outcome was changed in the nutritional indices from baseline till the end of the study.

Results

A total of 60 patients were randomly allocated to receive a low-protein diet supplemented with ketoacids (n=30) or control (n=30). All participants were included in the analysis of all outcomes. The mean change score in serum total protein, albumin, and triglycerides between the intervention and non-intervention groups were 1.1±1.1 g/dL vs 0.1±1.1 g/dL (p<0.001), 0.2±0.9 g/dL vs -0.3±0.8 g/dL (p<0.001), and 3.0±3.5 g/dL vs 1.8±3.7 g/dL, respectively.

Conclusion and recommendation

The use of low-protein diet supplemented with ketoacids improved the anthropometric and nutritional indices in patients with stage 3-5 CKD.

## Introduction

Chronic kidney disease (CKD) has increased in its global burden over the last few years, particularly in Nigeria with increasing use of non-steroidal anti-inflammatory drugs (NSAIDs) and 'skincare products' from unverified and unlicensed vendors as well as a higher prevalence of hypertension which all increases the incidence of CKD. CKD is defined as an abnormality in kidney function and structure that has health implications and has been present for three months and more [1]. CKD is one of the non-communicable diseases with increasing morbidity and mortality worldwide and thus a major public health concern [2].

More than 800 million people globally, or 10% of the overall population, suffer from chronic renal disease; CKD is more common in older individuals, women, racial minorities, and those with diabetes and high blood pressure [3]. In Nigeria, incidence using the Cockcroft-Gault equation was observed to range from 24.4% to 26%, while other studies using the Modification of Diet in Renal Disease (MDRD) showed a prevalence of 2.5% to 14.2% [4,5]. Another study using the Chronic Kidney Disease Epidemiology Collaboration (CKD-EPI) equation recorded a prevalence of 12% [6]. Most reviews in Nigeria are hospital-based, and patients who have poor access to hospital facilities are missed, resulting in under-reporting. Also, in Nigeria, health care is catered to by “out-of-pocket” payment, which most of the population cannot afford. The consequence of this health care system is that most patients tend to source for alternative forms of treatment and eventually presenting to the hospital with advanced kidney disease. Malnutrition is one of the complications of CKD, and when it is diagnosed and managed early, morbidity and mortality associated with CKD can be mitigated.

The indices used in assessing nutritional status in CKD patients are serum albumin, transferrin, pre-albumin, and retinol-binding protein, all of which assess visceral protein [7]. A low-protein diet has been the mainstay of managing malnutrition-related complications associated with CKD over the last 100 years. With the introduction of the very low protein diet supplemented with ketoacids, there have been some promising results. A low-protein diet or very low protein diet + ketoacids has been noticed to have an effect on the outcome of the nutritional status [7,8]. The main goal of a low-protein diet in CKD is to reduce the generation and accumulation of unexcreted nitrogenous waste, thereby reducing the complications associated with it, and at the same time maintaining adequate nutritional status of the patient [9,10].

Findings have shown the importance of low-protein diet in improving several renal outcomes, and supplementation with ketoacids also suggests specific improvement in nutritional indices [11-13].

## Materials and methods

The study was randomized, double-blind, and placebo-controlled. A total of 60 CKD stage 3-5 patients from Delta State University Teaching Hospital (DELSUTH), Oghara, Nigeria, consented to the study and met the inclusion criteria irrespective of etiological factors or clinic of recruitment. Ethical approval with approval number HREC/2018/058/0306 was granted by the Health Research Ethical Committee of DELSUTH. Hemodialysis-naïve patients were recruited from the renal clinic. The renal clinic holds on Mondays and Fridays and caters to an average of 30 patients every week.

The eligible study population of CKD patients stages 3-5 who consented to participate in the study had their anthropometric and biochemical parameters assessed at the beginning of the study and thereafter were advised to be on a low-protein diet of 0.6 g/kg/day for four weeks [14]. Participants were counselled at monthly clinic visits and on bi-weekly phone calls about dietary adherence, with the aim of achieving at least 50% dietary adherence. At the end of the four weeks, only anthropometric parameters were again assessed in the study population and then the participants were randomized into an intervention group and a non-intervention group. The intervention group received a low-protein diet in addition to ketoacid tablets (Nocid), while the non-intervention group continued with a low-protein diet supplemented with a placebo. The ketoacid prescription was four tablets daily in three divided doses taken during meals: two tablets in the morning, one tablet in the afternoon, and one tablet at night. Both groups were followed up for four months. At the end of the four-month follow-up period, biochemical parameters were repeated to assess for any change in nutritional indices [15]. The change in nutritional and biochemical indices was defined as the difference between the values at the end of the study (four months) and the baseline values. For this study, poor nutritional indices was defined using a combination of low serum albumin (<3.4 g/dL) and low serum cholesterol (<180 g/dL). The International Standard Classification of Occupations (ISCO-08), which is a four-level hierarchically structured classification, was used in grading occupation of participants; it is a model for the development of national and regional classifications of occupations. The four groups are major, sub-major, minor, and unit groups [16].

Data analysis

Anthropometric measurements and biochemical parameters collected were entered and analyzed using SPSS Version 20.0 (IBM Corp., Armonk, NY). Data were presented in tables, which were drawn using Microsoft Excel 2007. The full analysis set included patients who have received a low-protein diet supplemented with ketoacids versus a low-protein diet plus placebo, and data were analyzed based on the intention-to-treat principle and included all participants randomized in the study. Descriptive statistics was computed for each treatment group. Frequencies and percentages were used to represent categorical data such as sex, occupation, and level of education. Continuous variables such as age were represented as means and standard deviations. The chi-square test significance of Fisher’s exact was used to test for a difference in categorical variables, while the independent Student’s t-test was used to test for differences in the means of continuous variables between the two groups. The primary outcome and other continuous outcomes were assessed with a mean change score from baseline approach. The hypothesis was two-sided, and the level of statistical significance was set at a 95% confidence interval (p<0.05).

## Results

Socio-demographic characteristics of participants

A total of 60 patients were included in this study and equally distributed in a 1:1 ratio between the intervention group (30 participants) and non-intervention group (30 participants). The socio-demographic characteristics of the study participants are shown in Table 1. The overall mean age of the study participants was 47.5±11.9 years, and there was no difference in the mean age distribution between the two groups (p=0.7). There was a higher proportion of participants who were married, were Christian, and of level I occupation, with tertiary level education in both treatment groups. Individuals with a tertiary level of education comprised 50% of respondents (p=0.03).

**Table 1:** Baseline socio-demographic distribution of the study participants ‡Fishers exact, ¶independent t-test, †chi-square test, §Yates’ correction, *International Standard Classification of Occupation

Variable	Intervention (n=30), N (%)	No intervention (n=30), N (%)	Total, N (%)	Statistics test	P-value
Age categories (years)					
20-29	3 (10)	2 (6.7)	5 (8.3)	3.11‡	0.73
30-39	6 (20)	6 (20)	12 (20		
40-49	8 (26.7)	11 (36.7)	19 (31.7)		
50-59	7 (23.3)	9 (30)	16 (26.7)		
60-69	4 (13.3)	1 (3.3)	5 (8.3)		
70-79	2 (6.7)	1 (3.3)	3 (5)		
Mean age (years)	47.1±12.7	47.8±11.4	47.5±11.9	0.90¶	0.82
Sex					
Male	18 (60)	18 (60)	36 (60)	0.000†	1.00
Female	12 (40)	12 (40)	24 (40)		
Marital status					
Single	6 (20)	5 (16.7)	11 (18.3)	0.11†	0.74
Married	24 (80)	25 (83.3)	49 (81.7)		
Religion					
Christianity	29 (96.7)	25 (83.3)	54 (90)	1.667§	0.20
Islam	1 (3.3)	5 (16.7)	6 (10)		
Occupation*					
Level 1	13 (43.3)	15 (50)	28 (46.7)	1.51‡	0.70
Level 2	5 (16.7)	2 (6.7)	7 (11.7)		
Level 3	8 (26.7)	9 (30)	17 (28.3)		
Level 4	4 (13.3)	4 (13.3)	8 (13.3)		
Level of education					
Primary	9 (30)	2 (6.7)	11 (18.3)	7.03†	0.03
Secondary	6 (20)	13 (43.3)	19 (31.7)		
Tertiary	15 (50)	15 (50)	30 (50)		

Distribution of clinical variables among study participants

Table 2 shows the distribution of clinical categories in participants within the groups. The mean systolic blood pressure in the overall study population was 135.7±12.4 mmHg, while the diastolic blood pressure was 79.9±7.5 mmHg. Respondents with CKD for less than one year made up 53.3% (32 patients) of the study population, those with CKD stage 3 had the highest preponderance at 80% (25 patients), and those with stage 5 CKD were least represented at 3.3% (two patients). The commonest risk factor encountered was hypertension with a frequency of 46.7% (28), followed by glomerulonephritis (25%), systemic lupus erythematosus (18.3%), and diabetes (10%).

**Table 2:** Baseline clinical characteristics of study participants ¶Independent t-test, †Chi square, ‡Fishers exact, #Yates’ correction SBP, systolic blood pressure; DBP, diastolic blood pressure; CKD, chronic kidney disease; SLE, systemic lupus erythematosus

Variable	Intervention group	Non-intervention group	Mean±SD	Statistics test	P-value
Mean SBP (mmHg)	135.7±12.4	137.03±9.4	136.4±10.9	0.77¶	0.63
Mean DBP (mmHg)	80.8±9.2	79.0±5.3	79.9±7.5	0.41¶	0.32
Duration of CKD					
<1 year	15 (50)	17 (56.7)	32 (53.3)	0.27†	0.61
>1 year	15 (50)	13 (43.3)	28 (46.7)		
Stage of CKD					
Stage 3	23 (76.7)	25 (83.3)	48 (80)	3.25‡	0.20
Stage 4	7 (23.3)	3 (10)	10 (16.7)		
Stage 5	0 (0)	2 (6.7)	2 (3.3)		
Hypertension					
Yes	12 (40)	16 (53.3)	28 (46.7)	1.07†	0.33
No	18 (60)	14 (46.7)	32 (53.3)		
Diabetes mellitus					
Yes	3 (10)	3 (10)	6 (10)	0.000#	0.17
No	27 (90)	27 (90)	54 (90)		
Glomerulonephritis					
Yes	8 (26.7)	7 (23.3)	15 (25)	0.089†	1.00
No	22 (73.3)	23 (76.7)	45 (75)		
SLE					
Yes	7 (23.3)	4 (13.3)	11 (18.3)	1.002†	0.32
No	23 (76.7)	26 (86.7)	49 (81.7)		

Baseline nutritional indices of the study participants are shown in Table 3. 

**Table 3:** Baseline nutritional indices of the study participants

Biochemical parameters	Intervention (n=30)	Non-intervention (n=30)
Serum albumin	3.2±0.4	3.2±0.3
Serum total protein	6.6±0.5	6.6±0.6
Serum cholesterol	188.4±4.6	190.3±3.7
Serum triglycerides	130.2±6.4	129.4±6.3

Effects of intervention on nutritional indices

The mean serum albumin in the intervention group increased from baseline to the fourth month by +0.2±0.9 g/dL, while it decreased at the fourth month in the non-intervention group by -0.3±0.8 g/dL. The mean change score between the two groups was significant (p=0.001). The mean serum total protein levels increased from baseline in the intervention group but remained the same in the non-intervention group. The total serum protein showed a significant difference in the mean change scores between both groups (p=0.001) There was a significant change in the mean change score in the serum triglycerides in the intervention group when compared to the non-intervention group, while there was an increase in cholesterol from the baseline to the fourth month in both groups, with the increase in the intervention group being more; however, the mean change score was not significant between both groups (p=0.40), as shown in Table 4.

**Table 4:** Effects of low-protein diet supplemented with ketoacids on nutritional indices in participants

Parameter	Intervention, mean±SD	Non-intervention, mean±SD	P-value
Serum albumin (g/dL)			
Baseline	3.2±0.4	3.2±0.3	
Fourth month	3.4±0.4	2.9±0.3	
Mean change score	+0.2±0.9	-0.3±0.8	0.001
Serum total protein (g/dL)			
Baseline	6.6±0.5	6.6±0.6	
Fourth month	7.5±0.6	6.5±0.7	
Mean change score	+1.1±1.1	0.1±1.1	<0.001
Serum total cholesterol (mg/dL)			
Baseline	188.4±4.6	190.3±3.7	
Fourth month	190.1±2.7	191.7±3.9	
Mean change score	+1.7±2.7	+1.4±2.8	0.400
Serum triglycerides (mg/dL)			
Baseline	130.2±6.4	129.4±6.3	
Fourth month	133.2±5.8	131.2±7.1	
Mean change score	+3±3.5	+1.8±3.7	0.001

## Discussion

CKD is one of the non-communicable diseases with increasing morbidity and mortality worldwide and thus a major public health concern. It is defined as any abnormality in kidney function and structure that has health implications and has been present for three months and more [1,2].

This study assessed the effects of a low-protein diet supplemented with ketoacids versus a low-protein diet alone in CKD patients in stages 3-5. The emphasis of this study was on its effect on the nutritional indices: serum albumin, total protein, and triglycerides. The index study revealed a significant increase in serum albumin and total protein in the intervention group in comparison to the non-intervention group.

It was observed in this study that the mean serum albumin levels increased from baseline values in the ketoacid-supplemented group (intervention), while it decreased in the low-protein diet alone group (non-intervention). The mean change between both groups was statistically significant (<0.001). A similar study conducted in the Czech Republic reported an increase in serum albumin levels at the end of their study [17]. A neutral position was seen as the outcome of the study conducted by Feiten et al. [7], where no change in protein and albumin levels was reported. This may be because a very low-protein diet and ketoacids were used in the treatment arm of their study, and only advanced stage 5 CKD patients were recruited in their study, unlike this study that recruited patients in stages 3-5. The index study also noted a rise in serum total protein in the intervention group; this could be because leucine in the ketoacids reduced muscle breakdown and protein catabolism [18].

Cholesterol and triglycerides are other nutritional indices that were assayed in this study, and both indices increased throughout the study period but remained within the normal range in both study groups, Garneata and Mircescu [12] and Feiten et al. [7] both recorded no change in lipid profile levels in either treatment or non-treatment arms throughout their study duration. In contrast, the study carried out in the Czech Republic found a reduction in cholesterol and triglyceride levels in the treatment arm, and the study was carried out over three years [17]. A possible reason for the slight rise in both cholesterol and triglycerides may be that compliance with the low-protein diet was difficult and individuals probably compensated by increasing or eating more carbohydrates and other food sources. Another reason for the elevated levels of triglycerides observed in both groups in this study is that lipid abnormalities, particularly hypertriglyceridemia and hypercholesterolemia, are associated with CKD due to abnormal metabolism of lipoproteins.

Study limitation

This study is limited as the amount of low-protein diet taken by participants could not be measured or quantified.

## Conclusions

The results of this study support the beneficial effects of a low-protein diet supplemented with ketoacids in CKD patients on nutritional indices. This study also reported that the use of a low-protein diet supplemented with ketoacids may help reduce protein catabolism, thereby increasing total protein and albumin. From this study, it was also shown that the total level of serum triglycerides and cholesterol increased in the course of this study; however, this was within the normal ranges of serum cholesterol and triglycerides. A low-protein diet supplemented with ketoacids has shown benefits in CKD patients. There is a need for ketoacids to be more readily prescribed in CKD patients from stages 3 to 5. Physicians should also work more closely with dieticians, nutritionist, and patients and their relatives to find a more cost-effective way to ensure that CKD patients receive a low-protein diet of high biologic value.

## References

[1] KDIGO CKD Work Group (2013). KDIGO 2012 Clinical Practice Guideline for the Evaluation and Management of Chronic Kidney Disease. Kidney Int Suppl.

[2] Hecking E, Bragg-Gresham JL, Rayner HC (2004). Haemodialysis prescription, adherence and nutritional indicators in five European countries: results from the Dialysis Outcomes and Practice Patterns Study (DOPPS). Nephrol Dial Transplant.

[3] Koppe L, Cassani de Oliveira M, Fouque D (2019). Ketoacid analogues supplementation in chronic kidney disease and future perspectives. Nutrients.

[4] Chukwuonye II, Ogah OS, Anyabolu EN (2018). Prevalence of chronic kidney disease in Nigeria: systematic review of population-based studies. Int J Nephrol Renovasc Dis.

[5] Okwuonu CG, Chukwuonye II, Adejumo OA, Agaba EI, Ojogwu LI (2017). Prevalence of chronic kidney disease and its risk factors among adults in a semi-urban community of South-East Nigeria. Niger Postgrad Med J.

[6] Olanrewaju TO, Aderibigbe A, Popoola AA (2020). Prevalence of chronic kidney disease and risk factors in North-Central Nigeria: a population-based survey. BMC Nephrol.

[7] Feiten SF, Draibe SA, Watanabe R, Duenhas MR, Baxmann AC, Nerbass FB, Cuppari L (2005). Short-term effects of a very-low-protein diet supplemented with ketoacids in nondialyzed chronic kidney disease patients. Eur J Clin Nutr.

[8] Satirapoj B, Vongwattana P, Supasyndh O (2018). Very low protein diet plus ketoacid analogs of essential amino acids supplement to retard chronic kidney disease progression. Kidney Res Clin Pract.

[9] Schober-Halstenberg HJ (2005). Keto acid therapy in CKD patients epidemiology of CKD worldwide. Am J Nephrol.

[10] Aparicio M, Chauveau P, Précigout V, Bouchet JL, Lasseur C, Combe C (2000). Nutrition and outcome on renal replacement therapy of patients with chronic renal failure treated by a supplemented very low protein diet. J Am Soc Nephrol.

[11] Aparicio M, Bellizzi V, Chauveau P (2013). Do ketoanalogues still have a role in delaying dialysis initiation in CKD predialysis patients?. Semin Dial.

[12] Garneata L, Mircescu G (2013). Effect of low-protein diet supplemented with keto acids on progression of chronic kidney disease. J Ren Nutr.

[13] Oluseyi A, Enajite O (2016). Malnutrition in pre-dialysis chronic kidney disease patients in a teaching hospital in Southern Nigeria. Afr Health Sci.

[14] Kopple JD (2001). National kidney foundation K/DOQI clinical practice guidelines for nutrition in chronic renal failure. Am J Kidney Dis.

[15] Pendota S, Surabhineni SAK, Katnapally AS, Porandla D, Beemreddy SK (2017). Classification and applying pharmacovigilance principles to study adverse drug reaction and its 76 management. Int J Basic Clin Pharmacol.

[16] (2023). The International Standard Classification of Occupations- ISCO-08. https://isco-ilo.netlify.app/en/isco-08/.

[17] Teplan V, Schück O, Knotek A, Hajný J, Horácková M, Kvapil M (2003). Enhanced metabolic effect of erythropoietin and keto acids in CRF patients on low-protein diet: Czech multicenter study. Am J Kidney Dis.

[18] Kovesdy CP (2022). Epidemiology of chronic kidney disease: an update 2022. Kidney Int Suppl.

